# Specific Incorporation of Polyunsaturated Fatty Acids into the *sn*-2 Position of Phosphatidylglycerol Accelerates Photodamage to Photosystem II under Strong Light

**DOI:** 10.3390/ijms221910432

**Published:** 2021-09-28

**Authors:** Haruhiko Jimbo, Koki Yuasa, Kensuke Takagi, Takashi Hirashima, Sumie Keta, Makiko Aichi, Hajime Wada

**Affiliations:** 1 Department of Life Sciences, Graduate School of Arts and Sciences, The University of Tokyo, Tokyo 153-8902, Japan; yuasa_koki98@fra.go.jp (K.Y.); takagi-kensuke543@g.ecc.u-tokyo.ac.jp (K.T.); thirashima@cc.kyoto-su.ac.jp (T.H.); hwada@g.ecc.u-tokyo.ac.jp (H.W.); 2 Department of Biochemistry and Molecular Biology, Graduate School of Science and Engineering, Saitama University, Saitama 338-8570, Japan; 3 Department of Biological Chemistry, Chubu University, Kasugai 487-8501, Japan; keta@isc.chubu.ac.jp (S.K.); makiko@isc.chubu.ac.jp (M.A.)

**Keywords:** *Synechocystis*, photoinhibition, free fatty acids, phosphatidylglycerol, polyunsaturated fatty acids

## Abstract

Free fatty acids (FFAs) are generated by the reaction of lipases with membrane lipids. Generated polyunsaturated fatty acids (PUFAs) containing more than two double bonds have toxic effects in photosynthetic organisms. In the present study, we examined the effect of exogenous FFAs in the growth medium on the activity of photosystem II (PSII) under strong light in the cyanobacterium *Synechocystis* sp. PCC 6803 (*Synechocystis*). PUFAs but not monounsaturated fatty acids accelerated the rate of photodamage to PSII by inactivating electron transfer at the oxygen-evolving complex. Moreover, supplemented PUFAs were specifically incorporated into the *sn*-2 position of phosphatidylglycerol (PG), which usually contains C16 fatty acids at the *sn*-2 position in *Synechocystis* cells. The disruption of the gene for an acyl-ACP synthetase reduced the effect of PUFAs on the photoinhibition of PSII. Thus, the specific incorporation of PUFAs into PG molecules requires acyl-ACP synthetase and leads to an unstable PSII, thereby accelerating photodamage to PSII. Our results are a breakthrough into elucidating the molecular mechanism of the toxicity of PUFAs to photosynthetic organisms.

## 1. Introduction

Fatty acids (FAs) are essential components consisting of membrane lipids in living cells. Free fatty acids (FFAs) are produced by lipases that hydrolyze acyl chains from membrane lipids and become substrates used for the biosynthesis of storage compounds and plant hormones as well as acylation of proteins. FFAs have a broad diversity in terms of the chain length, number, and position of double bonds with *cis*/*trans* configuration. Such a wide spectrum has different effects on photosynthetic activities, as reported in *Synechocystis* sp. PCC 6803 (hereafter *Synechocystis*) [1]. A polyunsaturated FA (PUFA), α-linolenic acid (18:3^Δ9,12,15^), increased the sensitivity of photosystem II (PSII) to light-induced damage to photosynthesis (photoinhibition), which had toxic effects on cellular growth [1,2]. It also inhibited photosynthetic activity, and the inhibition was mediated by its incorporation into membrane lipids via the action of the acyl-acyl carrier protein (acyl-ACP) synthetase (AAS) [3,4,5]. However, the molecular mechanisms underlying the inhibitory effect of 18:3^Δ9,12,15^ or other PUFAs on photosynthesis are still unclear.

PSII uses light energy to oxidize water molecules to extract electrons and for producing oxygen. Under strong light, excess light energy damages PSII (photodamage). The damaged PSII is repaired by a rapid repair cycle (repair) that depends on protein synthesis. The rate of PSII photodamage can be observed in the presence of inhibitors of protein synthesis such as lincomycin or chloramphenicol [6,7,8]. PSII photodamage has been observed for more than 50 years [9]; however, the molecular mechanism of PSII photodamage is still under investigation. The acceptor/donor side hypothesis describes how the reactive oxygen species (ROS) produced by limitation of the photosynthetic electron acceptor/donor cause PSII photodamage [10]. Further studies have shown that ROS inhibits PSII repair rather than PSII photodamage [11,12,13]. Several studies using inhibitors of protein synthesis revealed that the rate of photodamage is associated with light intensity but not with the rate of photosynthetic electron transport [8,14]. The most recent hypothesis describes PSII photodamage as occurring in two steps: (1) corruption of the oxygen-evolving complex (OEC) by the absorption of blue/UV light and (2) inactivation of D1, a reaction center of PSII, by the absorption of visible light by chlorophylls (Chls) [14,15]. Recently, visible light was also found to induce corruption of the OEC [16].

In the present study, we examined the effect of various unsaturated FFAs on the photoinhibition of PSII by using chemical biological techniques. When PUFAs were incorporated into the *sn*-2 position of PG molecules via the action of AAS, PSII complexes were destabilized and PSII was inactivated without affecting electron transfer at the reaction center of PSII. Here, we report the specific action of PUFAs on PSII photodamage, mainly by corrupting the OEC via acylation of PG with PUFAs at the *sn*-2 position.

## 2. Results

### 2.1. PUFAs Accelerate Photodamage by Inhibiting Electron Transfer at the Oxygen-Evolving Complex

**Figure 1:**
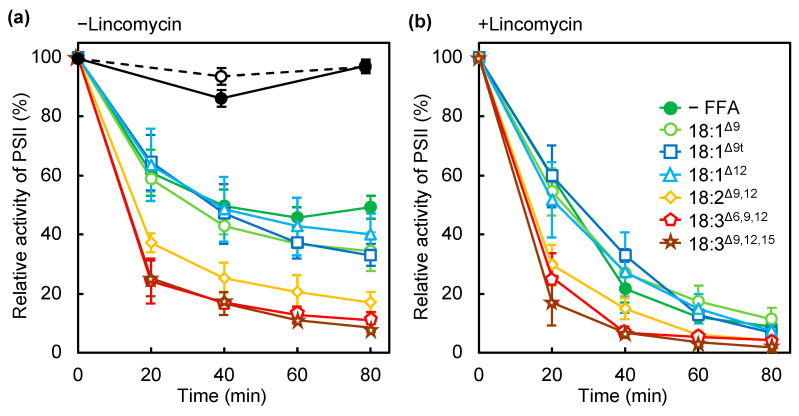
Effect of several C18 free fatty acids (FFAs) on the photoinhibition of PSII. Wild-type cells were incubated in the absence (filled green circles) or presence of oleic acid (18:1^Δ9^; light green circles), elaidic acid (18:1^Δ9t^; blue squares), *cis*-12-octadecenoic acids (18:1^Δ12^; light blue triangles), linoleic acid (18:2^Δ9,12^; yellow rhombuses), γ-linolenic acid (18:3^Δ6,9,12^; red pentagons), or α-linolenic acid (18:3^Δ9,12,15^; brown stars) at 32 °C under strong light at 1500 μmol photons m^−2^ s^−1^ under ambient aeration without (**a**) and with (**b**) lincomycin. Changes in PSII activity under weak light at 70 μmol photons m^−2^ s^−1^ under ambient aeration in the absence (empty circles) or presence (filled circles) of α-linolenic acid (18:3^Δ9,12,15^) are also shown in (**a**). FFAs were supplemented at a final concentration of 50 μmol L^−1^ just before illumination. The activity of PSII was monitored in terms of the evolution of oxygen in the presence of 1 mmol L^−1^ 1,4-benzoquinone as the electron acceptor. Values are the means ± SD of three independent experiments.

Unsaturated FFAs induce light-inducible photodamage in PSII, as we reported previously [1]. To further investigate the effect of unsaturated FAs on photosynthesis, we examined the effect of several unsaturated FFAs containing double bonds of different numbers, at different positions and with different configurations (*cis*/*trans*) on the photoinhibition of PSII (Figure 1a). Photodamage to PSII was observed in the presence of lincomycin, an inhibitor of protein synthesis that is required for the repair of PSII (Figure 1b). As we reported previously, 18:3^Δ9,12,15^, which has three *cis* double bonds at the Δ9, 12, and 15 positions, accelerated photoinhibition and photodamage to PSII, but oleic acid (18:1^Δ9^), with a *cis* double bond at the Δ9 position, did not (Figure 1). Other monounsaturated FAs (MUFAs), such as *cis*-12-octadecenoic acid (18:1^Δ12^), containing a *cis* double bond at Δ12, and elaidic acid (18:1^Δ9t^), containing a *trans* double bond at Δ9, did not accelerate the photodamage to PSII (Figure 1b). γ-linolenic acid (18:3^Δ6,9,12^) and linoleic acid (18:2^Δ9,12^) induced photoinhibition and photodamage to PSII to a similar extent to that induced by 18:3^Δ9,12,15^ (Figure 1), thus PUFAs but not *cis*- or *trans*-MUFAs induced photodamage to PSII. By contrast, under weak light, 18:3^Δ9,12,15^ did not affect the PSII activity. We chose 18:3^Δ9,12,15^ as a model PUFA and 18:1^Δ12^ for MUFA for the further experiments as described below. The content of these unsaturated FAs was very low in *Synechocystis* cells grown without FA supplementation, and thus, it was easy to trace them after incorporation into the cells.

**Figure 2:**
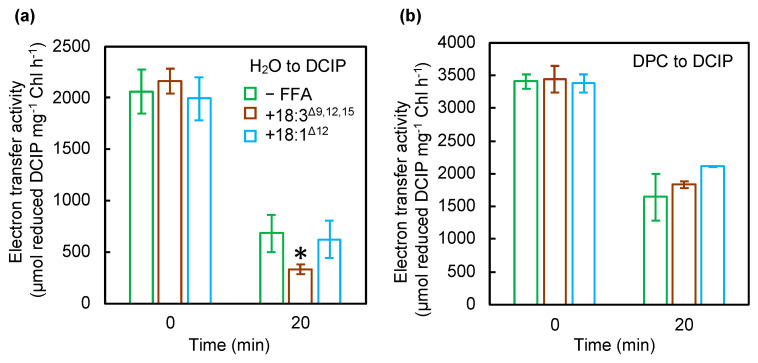
Effect of unsaturated C18 FFAs on electron transfer in PSII. Thylakoid membranes were prepared from cells exposed to strong light at 1500 μmol photons m^−2^ s^−1^ at 32 °C without (green bars) or with 18:3^Δ9,12,15^ (brown bars) or 18:1^Δ12^ (light blue bars) at a final concentration of 50 μmol L^−1^. The electron transfer activity from H_2_O (**a**) or diphenylcarbazide (DPC) (**b**) to 2,6-dichlorophenolindophenol (DCIP) was calculated by changes in absorbance at 580 nm due to the reduction of DCIP at 32 °C under strong light at 1500 μmol photons m^−2^ s^−1^. To measure the electron transfer rates from DPC to DCIP, the oxygen-evolving complex (OEC) in thylakoid samples was disrupted by incubation with 5 mM NH_2_OH for 5 min on ice. Values are the means ± SD (bars) of three independent experiments. Asterisks indicate statistically significant differences (* *p* < 0.05; Student’s *t*-test).

According to the two-step model, photodamage to PSII can occur in two steps: (1) corruption of the OEC and (2) inactivation of electron transfer in the reaction center of PSII [14,15]. We examined the effect of PUFAs on the electron transfer activity of PSII by using diphenylcarbazide (DPC) and 2,6-dichlorophenolindophenol (DCIP) as an electron donor and acceptor, respectively [17]. The activity of electron transfer from H_2_O or DPC to DCIP in the thylakoid membranes from wild-type (WT) cells incubated with or without 18:3^Δ9,12,15^ decreased under exposure to strong light for 20 min (Figure 2). Moreover, 18:3^Δ9,12,15^ inhibited the electron transfer activity from H_2_O to DCIP (Figure 2a), which corresponds to the whole PSII activity and is consistent with the results obtained in Figure 1a. The electron transfer activity from DPC to DCIP, which corresponds to the electron transfer within D1/D2, decreased at a similar rate to that in the thylakoid membranes from cells incubated without 18:3^Δ9,12,15^ (Figure 2b). When incubated with 18:1^Δ12^, the electron transfer activity from H_2_O or DPC to DCIP decreased at a similar rate to that in thylakoid membranes from cells incubated without 18:1^Δ12^ (Figure 2). Therefore, 18:3^Δ9,12,15^ accelerated the corruption of the OEC but not inactivation of electron transfer within the reaction center of PSII.

### 2.2. 18:3^Δ9,12,15^ Destabilizes Photosynthetic Complexes under Strong Light

**Figure 3:**
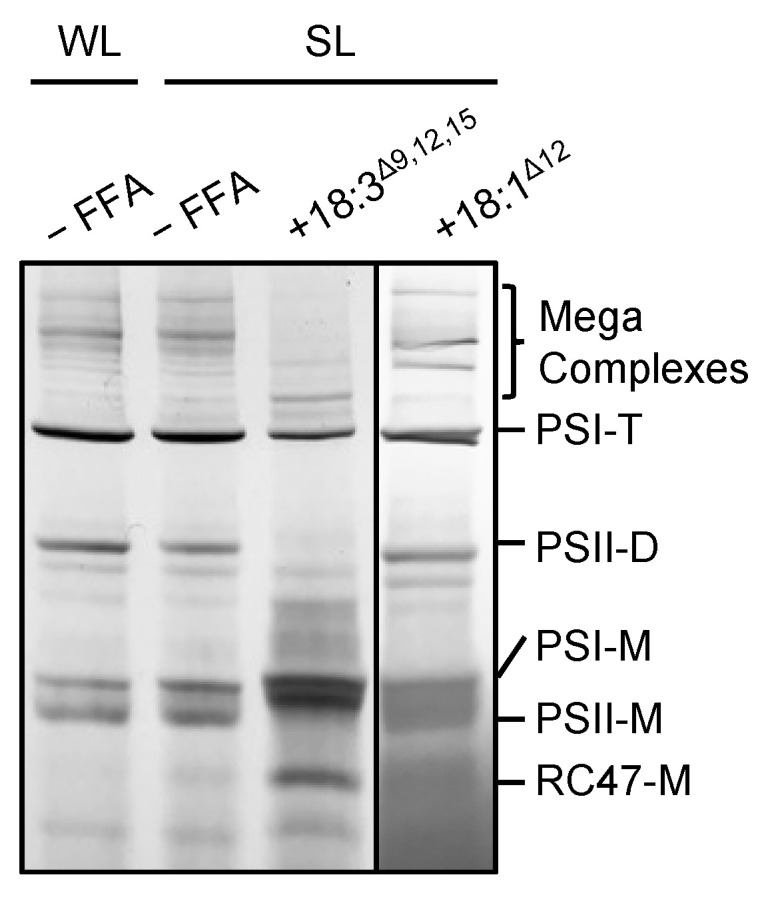
Effect of unsaturated C18 FFAs on photosynthetic complexes. Protein complexes in thylakoid membranes from cells incubated without (–FFA) or with 50 μmol L^−1^ 18:3^Δ9,12,15^ or 18:1^Δ12^ under weak light at 70 μmol photons m^−2^ s^−1^ (WL) or strong light at 1500 μmol photons m^−2^ s^−1^ (SL) for 20 min were solubilized by 1% (*w*/*v*) n-dodecyl-β-D-maltoside and separated by Blue-Native polyacrylamide gel electrophoresis. Bands on the gels were visualized by washing out excess Coomassie Brilliant Blue G-250 with distilled water. Bands for the photosynthetic complexes are identified as described previously [19]. PSI-T, Photosystem I trimer; PSII-D, Photosystem II dimer; PSI-M, Photosystem I monomer; PSII-M, Photosystem II monomer; RC47-M, CP43-less Photosystem II monomer.

To investigate the effect of 18:3^Δ9,12,15^ on photosynthetic complexes under strong light, we analyzed protein complexes in thylakoid membranes from cells treated with 50 μmol L^−1^ 18:3^Δ9,12,15^ under strong light (Figure 3). In cells treated without 18:3^Δ9,12,15^, exposure to strong light slightly decreased the amount of PSII dimer and PSI trimer, and increased PSI and PSII monomers (Figure 3). By contrast, in cells incubated with 18:3^Δ9,12,15^ under strong light, mega-complexes and the amount of PSI trimer and PSII dimer and monomer complexes decreased greatly, and the amount of PSI monomer and CP43-less PSII monomer complexes (RC47) increased (Figure 3). Therefore, 18:3^Δ9,12,15^ destabilized and dissociated photosystem complexes. However, 18:1^Δ12^ did not have a significant effect on the photosystem complexes under strong light (Figure 3). Several lipid molecules located between protein subunits of the photosystems function as glue to stabilize the tertiary structures [18]. Thus, the incorporation of PUFAs into membrane lipids might affect the stability of protein complexes in the thylakoid membrane, which would lead to disassembly and inactivation of the PSII complexes.

### 2.3. FFAs Are Incorporated into the sn-2 Position of PG Molecules by the Action of AAS under Strong Light

**Figure 4:**
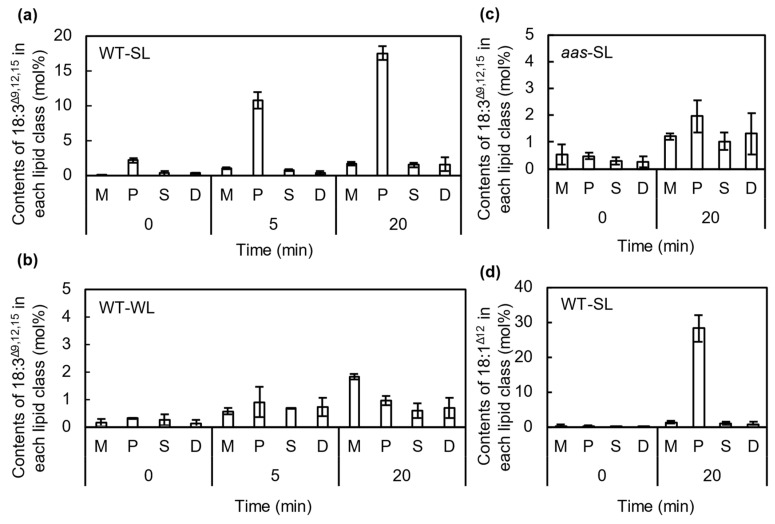
Specific incorporation of unsaturated C18 fatty acids into phosphatidylglycerol (PG) molecules via acyl-ACP synthase (AAS). The content of 18:3^Δ9,12,15^ incorporated into membrane lipids in wild-type (**a**) or *aas* cells (**c**) under strong light (SL) at 1500 μmol photons m^−2^ s^−1^ or weak light (WL) at 70 μmol photons m^−2^ s^−1^ (**b**) was analyzed by thin-layer chromatography (TLC) and gas chromatography (GC). M, MGDG; P, PG; S, SQDG; D, DGDG. Content of 18:1^Δ12^ incorporated into the membrane lipids under SL at 1500 μmol photons m^−2^ s^−1^ for 20 min was also analyzed by TLC and GC (**d**). Values are the means ± SD (bars) of three independent experiments.

To investigate the incorporation of 18:3^Δ9,12,15^ into membrane lipids, we analyzed the FA composition of membrane lipids from cells incubated with 50 μmol L^−1^ 18:3^Δ9,12,15^ under strong (Figure 4a) or weak light (Figure 4b). Because *Synechocystis* cells synthesize only a minor amount of 18:3^Δ9,12,15^ at 32 °C [20], its presence in membrane lipids was mostly due to the incorporation of exogeneous 18:3^Δ9,12,15^ in the culture medium into membrane lipids. Under strong light conditions, 18:3^Δ9,12,15^ was mainly detected in PG, so exogeneous 18:3^Δ9,12,15^ in the culture medium was mainly incorporated into PG. Under strong light, more than 18 mol% of the total FAs in PG was replaced with 18:3^Δ9,12,15^ during exposure to strong light for 20 min (Figure 4a). Although 18:3^Δ9,12,15^ was also incorporated into other lipids, such as monogalactosyldiacylglycerol (MGDG), sulfoquinovosyldiacylglycerol (SQDG), and digalactosyldiacylglycerol (DGDG), the proportions were very low: up to 2 mol% after 20 min of incubation (Figure 4a). Under weak light, a little 18:3^Δ9,12,15^ was incorporated into the membrane lipids without any specificity (Figure 4b).

**Figure 5:**
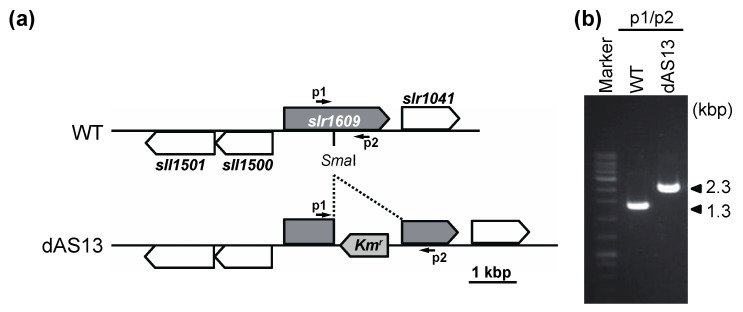
Construction of the *aas* insertional mutant (dAS13) of *Synechocystis*. (**a**) Diagrams showing the maps of the *aas* loci of the wild-type (WT) and the mutant (dAS13) *Synechocystis.* The arrows indicate the PCR primers p1 (5′-AAGGGGTGATGCTCAGCCACGG-3′) and p2 (5′-TTGGGTTACCACTGGTCGTTTGAGC-3′) used to screen for homozygous strains in the *aas* locus. The primers amplify 1.3 kb and 2.3 kb DNA fragments from the *Synechocystis* WT strain and the dAS13 mutant, respectively. (**b**) PCR analysis of the *aas* region of WT and dAS13. The primer pair p1/p2 was used for PCR and the products were analyzed by electrophoresis on a 1.0% agarose gel.

To check whether AAS is involved in the incorporation of 18:3^Δ9,12,15^ into PG under strong light, we determined the content of 18:3^Δ9,12,15^ in membrane lipids in *aas* mutant cells generated by insertion of the kanamycin-resistance gene cassette in the middle of the gene (Figure 5). When the mutant cells were incubated with 18:3^Δ9,12,15^ under strong light for 20 min, the specific incorporation of 18:3^Δ9,12,15^ into PG was much reduced (Figure 4c). We also checked the incorporation of 18:1^Δ12^, which did not have any effects on PSII photoinhibition (Figure 1a). *Synechocystis* cells do not contain 18:1^Δ12^ because a double bond at the Δ12 position is not introduced by the Δ12 desaturase before its introduction to the Δ9 position by Δ9 desaturase [21]. Therefore, its presence in membrane lipids indicates the incorporation of exogeneous 18:1^Δ12^ into membrane lipids. The 18:1^Δ12^ was mainly detected in PG (Figure 4d), so 18:1^Δ12^ is also specifically incorporated into PG.

**Figure 6:**
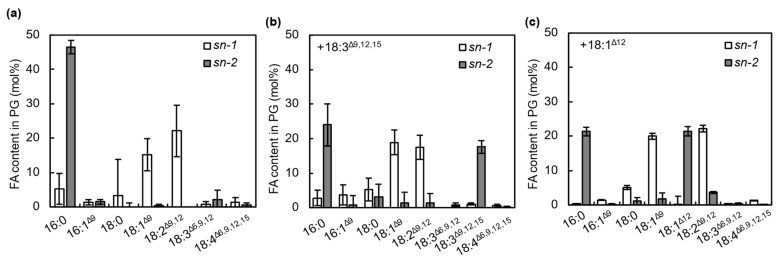
Distribution of incorporated unsaturated C18 FAs in PG molecules. PG molecules prepared from cells incubated without FA (**a**) and with 50 μmol L^−1^ 18:3^Δ9,12,15^ (**b**) or 18:1^Δ9^ (**c**) under strong light at 1500 μmol photons m^−2^ s^−1^ for 20 min were treated with honey bee phospholipase A_2_, and the resulting 1-acyl-lysoPGs were analyzed by thin-layer chromatography and gas chromatography to determine the composition of fatty acids at the *sn*-1 position of PG. The composition of fatty acids at the *sn*-2 position was calculated by subtracting the fatty acid composition at the *sn*-1 position from the total fatty acid composition. Values are the means ± SD (bars) of three independent experiments.

*Synechocystis* PG molecules usually contain C18 FAs at the *sn*-1 position and C16 FAs at the *sn-2* position (Figure 6a) [20,22]. We checked whether 18:1^Δ12^ and 18:3^Δ9,12,15^ were incorporated into the *sn*-1 or 2 position of PG molecules by treating purified PG from FA-treated *Synechocystis* cells with honey bee phospholipase A_2_, which specifically cleaves FAs at the *sn*-2 position of PG molecules. Most of the 18:1^Δ12^ and 18:3^Δ9,12,15^ was detected at the *sn*-2 position but not the *sn*-1 position of PG molecules (Figure 6b,c). Therefore, 18:1^Δ12^ and 18:3^Δ9,12,15^ were specifically incorporated into the *sn*-2 position of PG by the action of AAS. Our results are consistent with the finding that FAs in PG molecules are remodeled in *Synechocystis* cells [23]. These results suggest that the incorporation of PUFAs into the *sn*-2 position of PG destabilizes photosystems, as shown in Figure 3, and enhances the photodamage to PSII.

### 2.4. Disruption of the Gene for AAS Protects PSII against PUFA-Induced Photoinhibition

**Figure 7:**
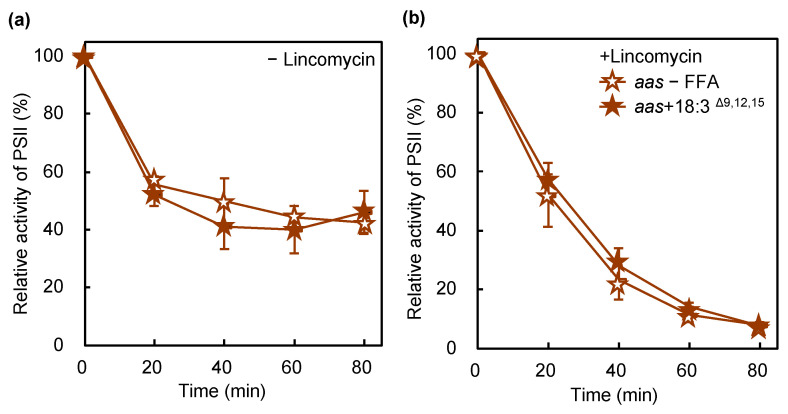
Effect of 18:3^Δ^^9,12,15^ on the photoinhibition of PSII in an AAS-lacking mutant. *aas* cells in the absence (brown empty stars) or presence (brown filled stars) of 50 μmol L^−1^ 18:3^Δ9,12,15^ were incubated at 32 °C under light at 1500 μmol photons m^−2^ s^−1^ with ambient aeration in the absence (**a**) and presence (**b**) of lincomycin. The 18:3^Δ9,12,15^ was supplemented at a final concentration of 50 μmol L^−1^ just before the onset of illumination. The activity of PSII was monitored in terms of the evolution of oxygen in the presence of 1 mmol L^−1^ 1,4-benzoquinone as the electron acceptor. Values are the means ± SD (bars) of three independent experiments.

Disruption of the gene for AAS induces tolerance in cells to the PUFA-induced inhibition of cellular growth [3,4]. To examine the role of AAS in PUFA-induced PSII photoinhibition, we performed similar experiments with a mutant lacking AAS. AAS was required for the incorporation of 18:3^Δ9,12,15^ into membrane lipids, especially PG (Figure 4c). In wild-type cells, PSII activity dropped to 8% of the initial activity after the 80 min exposure to strong light in the presence of 18:3^Δ9,12,15^ (Figure 1a). AAS mutant cells had 46% PSII activity in the same time (Figure 7a), indicating that the lack of AAS reduced the effect of 18:3^Δ9,12,15^ on PSII photoinhibition or photodamage (Figure 7). Therefore, the incorporation of PUFAs into PG mediated by the action of AAS accelerates photodamage to PSII. In *Synechococcus elongatus* PCC 7942, the lack of AAS accelerated the photoinhibition of PSII [24]. A mutant lacking AAS had 42% of the initial PSII activity after 80 min of exposure to strong light, whereas wild-type cells retained 50% under the same conditions (Figure 1a and Figure 7a). Thus, the lack of AAS in *Synechocystis* slightly accelerated the photoinhibition of PSII as well. However, the effect was smaller than that observed in *Synechococcus elongatus* PCC 7942 under these conditions.

## 3. Discussion

### 3.1. Effect of PUFAs on Photodamage to PSII

In the present study, the specific incorporation of 18:3^Δ9,12,15^ into the *sn*-2 position of PG molecules increased the sensitivity of PSII to photodamage by destabilizing the PSII complexes. A mutant of *Synechocystis* lacking two lysophosphatidic acid acyltransferases (LPAATs) contained a high amount of C18 FAs at the *sn*-2 position in all membrane lipids and shows increased PSII sensitivity to strong light as compared with WT cells [25]. Hence, C16 FAs at the *sn*-2 position of membrane lipids are important. Our results also indicated the importance of C16 FAs at the *sn*-2 position of PG. A marine cyanobacterium, *Synechococcus* sp. PCC 7002 (hereafter *Synechococcus*), that lacked a Δ6 desaturase showed extreme sensitivity to PUFAs [2]. A transgenic strain of *Synechococcus* overexpressing *desD* for Δ6 desaturase (DesD) from *Synechocystis* generated 18:3^Δ6,9,12^ and 18:4^Δ6,9,12,15^, and grew better than the WT [26]. *Synechocystis* DesD introduces a double bond into fatty acids bound to galactolipids rather than those bound to SQDG and PG [27,28]; thus, the severe toxicity of PUFAs for *Synechococcus* might be due to the incorporation of PUFAs into PG molecules but not galactolipids. PG has crucial roles in the function and maintenance of PSII [29,30,31,32]. PG is also required for the oligomerization of both the PSI and PSII complexes [33,34] and for the binding of extrinsic proteins stabilizing the OEC in PSII [35]. More than 30 mol% of the total PG in the thylakoid membranes is located in the photosystems [36]. Therefore, unusual PG molecules containing PUFAs at the *sn*-2 position might specifically disorder the photosystems, especially the PSII complexes, with the resulting inactivation of PSII.

In bacteria, PG is used as a substrate for the lipidation of lipoproteins [37]. In cyanobacteria, several lumenal proteins such as CyanoP and CyanoQ, homologs of PsbP and PsbQ in land plants, are modified by diacylglycerol and palmitate [38]. CyanoP is required for stabilizing the OEC [39], and a lack of CyanoQ protein destabilizes PSII [40]. These observations suggest that unusual PG containing PUFAs at the *sn*-2 position becomes a substrate for lipidation of these lipoproteins, which might affect the stability of the OEC. A recent study by Knoppová et al. revealed that a PSII assembly factor, Ycf48, was lipidated by PG [41]. Therefore, the unstable PSII complex in the presence of PUFAs shown in Figure 3 might be due to the lipidation of Ycf48 by the unusual PG molecules.

### 3.2. Incorporation of PUFAs into PG

We showed that the effect of PUFAs on photosynthesis depends on the specific incorporation of PUFAs into the *sn*-2 position of PG molecules, which is mediated by AAS. *Synechocystis* AAS reacts nonspecifically with FFAs to synthesize acyl-ACPs in vitro [5]. These findings suggest that *Synechocystis* has a phospholipase A_2_ that synthesizes 1-acyl-lysoPG (lysoPG) by digesting an acyl group at the *sn*-2 position of PG, and that a lysoPG acyltransferase (LPGAT) transfers PUFAs into the *sn*-2 position of 1-acyl-lysoPG from ACP-attached PUFAs. Weier et al. (2005) identified an LPGAT that transferred both the C16- and C18-acyl groups from acyl-ACP but not acyl-CoA to lysoPG [42]. Therefore, this lysoPG acyltransferase might be responsible for the specific incorporation of PUFAs into the *sn*-2 position of PG molecules. However, tolerance to PUFAs in the mutant lacking LPGAT has not been investigated. *Synechocystis* has a phospholipase encoded by *sll1969* [43], which suggests that lysoPG is synthesized by the phospholipase in *Synechocystis* cells. Further biochemical analysis of the phospholipase is needed to investigate whether this phospholipase is involved in the synthesis of 1-acyl-lysoPG. *Synechococcus elongatus* PCC 7942, a mutant lacking AAS, produced more palmitic acid (16:0) and lyso-lipids under strong light [24]. This finding supports our result that the specific incorporation of PUFAs into PG molecules was accelerated under strong light.

AAS is broadly conserved among oxygenic photosynthetic organisms such as cyanobacteria and land plants [5]. PUFAs are toxic to other photosynthetic organisms as well [44], so the uptake of PUFAs into membrane lipids mediated by the action of AAS might cause a universal effect of PUFAs in photosynthetic organisms.

In plants, PG in the chloroplasts is exclusively synthesized inside the chloroplasts by the plastid pathway, which results in PG molecules containing 16:0 or Δ3-*trans*-hexadecenoic acid (16:1^Δ3t^) at the *sn*-2 position owing to the substrate specificity of LPAAT [45,46] and the desaturation catalyzed by desaturase (FAD4) [47] in the chloroplasts. However, glycolipids in the chloroplasts, such as MGDG, DGDG, and SQDG, are synthesized not only by the plastid pathway but also the endoplasmic reticulum (ER) pathway [48,49]. In the ER pathway, FAs synthesized in the chloroplasts are exported to the cytosol and used for the synthesis of phospholipids in the ER, then some synthesized phospholipids in the ER are transferred to the chloroplasts and used for the synthesis of glycolipids. The glycolipids synthesized through the ER pathway contain C18 PUFAs at the *sn*-2 position owing to the substrate specificity of LPAAT and the action of desaturases (FAD2 and FAD3) in the ER [50,51,52]. Our results suggest that PG needs to be synthesized by the plastid pathway to avoid the synthesis of PG molecules containing PUFAs at the *sn*-2 position because such PG molecules destabilize the PSII complex and accelerate the photoinhibition of PSII.

## 4. Materials and Methods

### 4.1. Strains and Culture Conditions

Cells of glucose-tolerant *Synechocystis* GT-I as WT cells were grown photoautotrophically in BG-11 medium at 32 °C, as described in [1]. Cells in the culture with an optical density at 730 nm of 1.0 ± 0.1 (about 3.6 μg mL^−1^ Chl *a*) were used for the assays. Generation of a mutant lacking AAS was as described in [4].

### 4.2. Targeted Inactivation of aas in Synechocystis

The *aas* gene (*slr1609*) of *Synechocystis* was amplified by PCR using TaKaRa LA Taq DNA polymerase (TAKARA BIO INC., Japan) and the primer pair a1 (5′-ACGCTTTGGTGATGAACACTGG-3′)/a2 (5′-TTGGCGTAGGGGAATGGCT-3′). The amplified DNA fragment was cloned into the pGEM-T easy vector (Promega, Madison, WI, USA). A 1.0-kb DNA fragment carrying the *npt*I kanamycin resistance gene was excised from the plasmid pRL250 [53] and ligated into the *Sma*I recognition site in the cloned *aas* gene. The resultant plasmid was used to transform the wild-type *Synechocystis* cells through homologous recombination into the kanamycin resistance type. After three rounds of streak purification of single colonies, the absence of the wild-type *aas* copy in selected colonies was confirmed by PCR. The resulting mutant, which carried an insertionally inactivated *aas* gene, was named dAS13 (Figure S1).

### 4.3. Photosynthetic Activity of PSII

PSII activity was measured by oxygen evolution from PSII in the presence of 1 mM 1,4-benzoquinone (BQ) and 1 mM K_3_Fe(CN)_6_, as described in [1]. For the analysis of photoinhibition of PSII, cells in the culture were exposed to strong light at 1500 μmol photons m^−2^ s^−1^ at 32 °C under ambient aeration. For assaying photodamage, the cell culture was supplemented with 200 μg mL^−1^ lincomycin before illumination. FFA compounds purchased from FujiFilm Wako (Tokyo, Japan) and Tokyo Chemical Industry (Tokyo, Japan) were added to the cell culture just before illumination.

To determine the electron transfer in PSII, we measured the rate of the photoreduction of DCIP, which accepts electrons from Q_B_, with isolated thylakoid membranes in the absence or presence of diphenylcarbazide (DPC), an electron donor to a tyrosine Z in PSII. Electron transfer rates from H_2_O or DPC to 2,6-dichlorophenolindophenol (DCIP) were measured as described in [17] with some modifications. Thylakoid membranes were isolated from cells incubated under strong light at 1500 μmol photons m^−2^ s^−1^ for 20 min with or without 50 μmol L^−1^ 18:3^Δ9,12,15^ or 18:1^Δ12^, then the cells were disrupted by bead-beating in Solution 8 (0.4 mol L^−1^ sucrose, 2 mmol L^−1^ MgCl_2_, 5 mmol L^−1^ NaCl, 40 mmol L^−1^ Mes-NaOH, pH 6.5). To measure the electron transfer rate from DPC to DCIP, thylakoid membranes were treated with 5 μmol L^−1^ NH_2_OH for 5 min on ice. NH_2_OH-treated thylakoid membranes were washed twice, then resuspended in Solution 8. The light-induced decrease in the absorbance by DCIP at 580 nm in the thylakoid membrane suspension (5 μg Chl mL^−1^) in the presence of 80 mmol L^−1^ DCIP and 80 μmol L^−1^ DPC under strong light at 1500 μmol photons m^−2^ s^−1^ at 32 °C for 5 min was measured by spectrophotometry (V-730Bio, JASCO, Tokyo, Japan).

### 4.4. Analysis of Photosynthetic Complexes by Blue-Native Polyacrylamide Gel Electrophoresis (PAGE)

Thylakoid membranes were isolated from WT cells disrupted by bead-beating with glass beads in Buffer A (50 mmol L^−1^ MES-NaOH pH 6.0, 10 mmol L^−1^ MgCl_2_, 5 mmol L^−1^ CaCl_2_, 25% glycerol), as described in [54]. Thylakoid membranes corresponding to 8 μg Chl (1 mg Chl mL^−1^) were solubilized with 1% (*w*/*v*) n-dodecyl-β-D-maltoside (β-DM) (Dojindo, Japan) for 20 min on ice. Protein complexes were separated on 4–20% Blue-Native-PAGE (ThermoFisher, Waltham, MA, USA) under a constant 5 mA for 14 h in a cold room. Excess Coomassie Brilliant Blue (CBB) G-250 was washed out with distilled water.

### 4.5. Lipid Analysis

To analyze the incorporation of 18:3^Δ9,12,15^ into membrane lipids, cells were incubated with 50 μmol L^−1^ 18:3^Δ9,12,15^ (Wako, Japan) under the same conditions as the analysis of PSII photoinhibition described above. A 5 mL aliquot of the cell culture was removed at the designated time and kept on ice until lipid extraction. Lipids in *Synechocystis* cells were extracted as described in [31]. Each membrane lipid was separated and isolated on a thin-layer chromatography (TLC) plate (TLC silica gel 60; Merck, Kenilworth, NJ, USA) developed with chloroform:methanol: 28% NH_4_OH = 65:35:5 (*v*/*v*/*v*), and FAs binding to each sugar lipid were methyl-esterified with HCl–methanol at 85 °C for more than 3 h. Since FFA is located at the same position as PG in TLC (Supplementary Figure S1), PG was methyl-esterified with 2 M KOH-methanol at room temperature for 2 min, which only methyl-esterified glycerolipids but not FFA, as described previously [55]. The obtained FA methylesters were analyzed by gas chromatography (GC) (GC-2014, Shimazu, Tokyo, Japan). To determine the content of FAs at the *sn*-1 and -2 positions of PG, PG isolated from cells treated with 50 μmol L^−1^ 18:3^Δ9,12,15^ for 20 min under strong light was treated with 0.5 μg honey bee phospholipase A_2_ (Sigma-Aldrich, St. Louis, MO, USA) in a lipase buffer (50 mmol L^−1^ Tris–HCl pH 7.5 with 0.05% (*w*/*v*) Triton X-100) for 30 min at 37 °C. The resulting FFAs and 1-acyl-lyso-phosphatidylglycerol (LysoPG) were separated on a TLC plate developed with chloroform:acetone:methanol:acetic acid:water = 50:20:10:15:5 (*v*/*v*/*v*/*v*/*v*), and FAs in 1-acyl-lysoPG was methyl-esterified with HCl–methanol at 85 °C for 3 h. The obtained FA methylesters were analyzed by GC (GC-2014, Shimadzu). FA content at the *sn*-2 position of the PG molecules was calculated by subtracting the FA content at the *sn*-1 position from the total fatty acid composition; 15:0 (5 nmol) was used as the internal control.

## Data Availability

The data supporting the findings of this study are available within the article.

## Supplementary Materials

Supplementary materials can be found at https://www.mdpi.com/article/10.3390/ijms221910432/s1.
