# Supplementary material for: Specific Incorporation of Polyunsaturated Fatty Acids into the sn-2 Position of Phosphatidylglycerol Accelerates Photodamage to Photosystem II under Strong Light
Source: Int J Mol Sci. 2021 Sep 28;22(19):10432. doi: 10.3390/ijms221910432 (PMC8508968; doi:10.3390/ijms221910432)
Supplement: Supplementary file 1 [file ijms-22-10432-s001.zip › ijms-1375710-supplementary.pdf]

Supplementaly figure S1

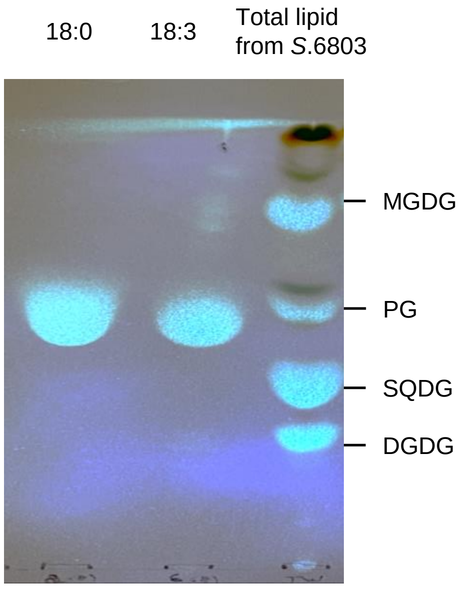

Figure S1. Position of free fatty acids on TLC developed with chloroform : methanol : 28% NH_4OH = 65 : 35 : 5 (v/v/v). 18:0, 18:3 and purified lipids from *Synechocystis* were applied to TLC plate that developed with chloroform : methanol : 28% NH_4OH = 65 : 35 : 5 (v/v/v). Lipids on TLC plate were visualized by primulin.
